# Role of L-Arginine supplementation in Long Covid-related Fatigue and Depression in Elderly Outpatients

**DOI:** 10.1192/j.eurpsy.2024.257

**Published:** 2024-08-27

**Authors:** G. Moniello, I. Bonfitto, F. Simonetti, S. Dimalta, M. R. Rizzo, A. Bellomo

**Affiliations:** ^1^Department of Geriatric Medicine and Gerontology, ASL CE, Caserta; ^2^Department of Mental Health, ASL FG, Foggia; ^3^Department of Geriatric and Internal Medicine, AOU Luigi Vanvitelli, Caserta; ^4^Department of Clinical and Experimental Medicine, University of Foggia, Foggia, Italy

## Abstract

**Introduction:**

Chronic fatigue and psychiatric manifestations (depression, anxiety and sleep disturbances) appear to be key features of post-COVID-19 syndrome and increase significantly in prevalence over time (Lavienraj et al. J Neurol Sci 2022;434:120162). Several studies have suggested an association between altered levels of arginine metabolites and depression, anxiety and stress severity (Arisoy et al. J Psychiatr Res 2020;120:21-28). L-arginine supplementation has also been shown to improve walking performance, muscle strength, endothelial function and fatigue in adults with Long COVID (Tosato et al. Nutrients 2022;14(23):4984).

**Objectives:**

To study effects of L-arginine oral supplementation on chronic fatigue and depressive symptoms reported 3 months or more after acute COVID-19 onset in elderly outpatients without severe comorbid conditions.

**Methods:**

This is a parallel-group, double-blind, randomized controlled trial conducted on 96 over 65 non-hospitalized patients suffering from Long Covid-related fatigue and depression. The first group included patients that received 1,66 g L-arginine twice a day in addition to a standard antidepressant therapy based on Selective Serotonin Reuptake Inhibitors (SSRIs), whereas the second group received antidepressant only. Severity of fatigue and depressive symptoms was evaluated at baseline and after 8 weeks of treatment using Fatigue Symptom Inventory (FSI) and Hamilton Rating Scale for Depression (HAM-D), respectively.

**Results:**

At baseline, 64 patients (66,7%) reported moderate fatigue (4-6) and the remaining 32 (33,3%) reported severe fatigue (7-10). In this phase the average HAM-D score was 12,85 ± 5,97; among patients, 57,3% experienced mild symptoms of depression, 32,3% experienced moderate symptoms and 6,4% experienced severe symptoms. After two months, patients treated with L-arginine supplementation exhibited a 30% greater improvement in fatigue-related symptom severity (p=0.008) and a significantly decrease in average HAM-D score (p=0.002) compared to the group treated with SSRI only.

**Conclusions:**

According to our results, adding oral L-arginine to standard antidepressant therapy in elders with Long Covid-related fatigue and depression significantly decreases severity of both physical and affective symptoms. Further studies are needed to clarify the intriguing role of L-arginine in the treatment of Post Covid-19 syndrome and its potential effects in promoting geriatric patients’ health, wellbeing and quality of life.

**Disclosure of Interest:**

None Declared

